# Long-term Progression and Risk Factors of Fundus Tessellation in the Beijing Eye Study

**DOI:** 10.1038/s41598-018-29009-1

**Published:** 2018-07-13

**Authors:** Yan Ni Yan, Ya Xing Wang, Yan Yang, Liang Xu, Jie Xu, Qian Wang, Xuan Yang, Jing Yan Yang, Wen Jia Zhou, Wen Bin Wei, Jost B. Jonas

**Affiliations:** 1 Beijing Tongren Eye Center, Beijing key Laboratory of Intraocular Tumor Diagnosis and Treatment, Beijing Ophthalmology & Visual Sciences Key Lab, Beijing Tongren Hospital, Capital Medical University, Beijing, China; 2 Beijing Institute of Ophthalmology, Beijing Tongren Eye Center, Beijing Tongren Hospital, Capital Medical University, Beijing Ophthalmology and Visual Science Key Lab, Beijing, China; 3 Beijing Aier-Intech Eye Hospital, Beijing, China; 4 Department of Ophthalmology, Medical Faculty Mannheim of the Ruprecht-Karls-University, Seegartenklinik, Heidelberg, Germany

## Abstract

To assess the progression of fundus tessellation, color fundus photographs of the participants of the longitudinal population-based Beijing Eye Study were examined. The study included 4439 subjects in 2001 and 2695 (66.4% of the surviving) individuals in 2011. Larger progression in macular fundus tessellation (mean: 0.24 ± 0.48 grades) was associated (multivariate analysis; correlation coefficient r: 0.53) with thinner subfoveal choroidal thickness in 2011 (*P* < 0.001; standardized regression coefficient beta: −0.37), older age (*P* < 0.001; beta: 0.22), higher level of education (*P* < 0.001; beta: 0.09), more myopic change in refractive error (*P* < 0.001; beta: −0.09) and lower cognitive function score (*P* = 0.02; beta: −0.05). Larger increase in peripapillary fundus tessellation (mean: 0.19 ± 0.26 grades) correlated with thinner peripapillary choroidal thickness in 2011 (*P* < 0.001; beta: −0.35), older age (*P* < 0.001; beta: 0.20), worse best corrected visual acuity (*P* = 0.001; beta: 0.07), more myopic change in refractive error (*P* < 0.001; beta: −0.07) and higher prevalence of ever smoking (*P* = 0.004; beta: 0.05). The increase in macular fundus tessellation, as a surrogate for thinning of the posterior choroid, was associated with lower cognitive function, after adjusting for choroidal thickness, age, educational level and change in refractive error. The findings point to the clinical value of the assessment of fundus tessellation and suggest potential associations between cognitive function and fundus tessellation/choroidal thickness.

## Introduction

Fundus tessellation has been defined as the visibility of large choroidal vessels at the posterior fundus pole outside of the peripapillary region^1–9^. In a previous cross-sectional population-based study, a higher degree of fundus tessellation was strongly associated with thinner subfoveal choroidal thickness^9^. When divided into 4 grades, the degrees of fundus tessellation differed markedly in subfoveal choroidal thickness (subfoveal choroidal thickness in fundus tessellation grade 0: 322 ± 90 µm; grade 1: 229 ± 80 µm; grade 2: 122 ± 52 µm; grade 3: 81 ± 37 µm). Also in multivariate analysis, a higher degree of fundus tessellation was strongly correlated with thinner subfoveal choroidal thickness, after adjusting for older age, male sex, lower body mass index, worse best-corrected visual acuity, longer axial length, larger parapapillary beta zone and higher glaucoma prevalence, and a lower prevalence of intermediate and late age-related macular degeneration^9^. It indicated that the degree of fundus tessellation might be taken as a surrogate for subfoveal choroidal thickness, if measurements of choroidal thickness were not available.

Previous studies have revealed the importance of choroidal thickness in the diagnosis of macular and other retinal diseases. To cite examples, an abnormally thick subfoveal choroid was associated with central serous choroidopathy, Vogt-Koyanagi-Harada’s disease, polypoidal choroidal vasculopathy and idiopathic choroidal neovascularization. An abnormally thin choroid was related with high myopia, to name only few examples^10–13^. Since the technique to measure subfoveal choroidal thickness has been available so far only for a few years, it has not yet been possible to longitudinally assess long-term changes in subfoveal choroidal thickness and its associations with the prevalence of ocular diseases^14–19^. We therefore determined the degree of fundus tessellation as a surrogate for subfoveal choroidal thickness, assessed changes in the degree of fundus tessellation over a 10-year period, and examined associations between the change in the degree of fundus tessellation and the prevalence of ocular disorders and systemic parameters. To reduce the risk of a referral bias, we choose a population-based recruitment of the study participants.

## Methods

The Beijing Eye Study is a population-based longitudinal study which was performed in a rural region and an urban region of Beijing. Its baseline examination was carried out in 2001, with two follow-up examinations conducted in 2006 and in 2011. According to the Declaration of Helsinki, the Medical Ethics Committee of the Beijing Tongren Hospital approved the study and all participants gave informed written consent. The ethics committee confirmed that all methods were performed in accordance with the relevant guidelines and regulations. In the baseline examination in 2001, 4403 individuals participated with 1633 (47.1%) individuals coming from the rural region and 1835 (52.9%) subjects living in the urban region.

All study participants underwent an interview with standardized questions on their socioeconomic background, quality of life, depression, physical activity, known major systemic diseases and quality of vision. They also underwent a medical and detailed ophthalmological examination. The latter included measurement of visual acuity, tonometry, digital photography of the cornea, lens, optic disc and macula, slit lamp assisted biomicroscopy of the anterior and posterior ocular segment, ocular biometry and spectral domain optical coherence tomography (Spectralis, Heidelberg Engineering Co., Heidelberg, Germany). The concentrations of glucose, glycosylated hemoglobin HbA1c, blood lipids creatinine and C-reactive protein were measured in blood samples taken under fasting conditions. We measured the blood pressure, body height and weight, and the circumference of waist and hip. For study purposes, we diagnosed diabetes mellitus as a fasting plasma glucose concentration ≥7.0 mmol/L or by a self-reported history of physician diagnosis of diabetes mellitus. Arterial hypertension was defined as systolic blood pressure ≥140 mm Hg and/or diastolic blood pressure ≥90 mm Hg and/or self-reported current treatment for arterial hypertension. Cognitive function was assessed using the Mini Mental Status Examination scale^20^. The study design has been described in detail previously^13,21^.

**Figure 1:**
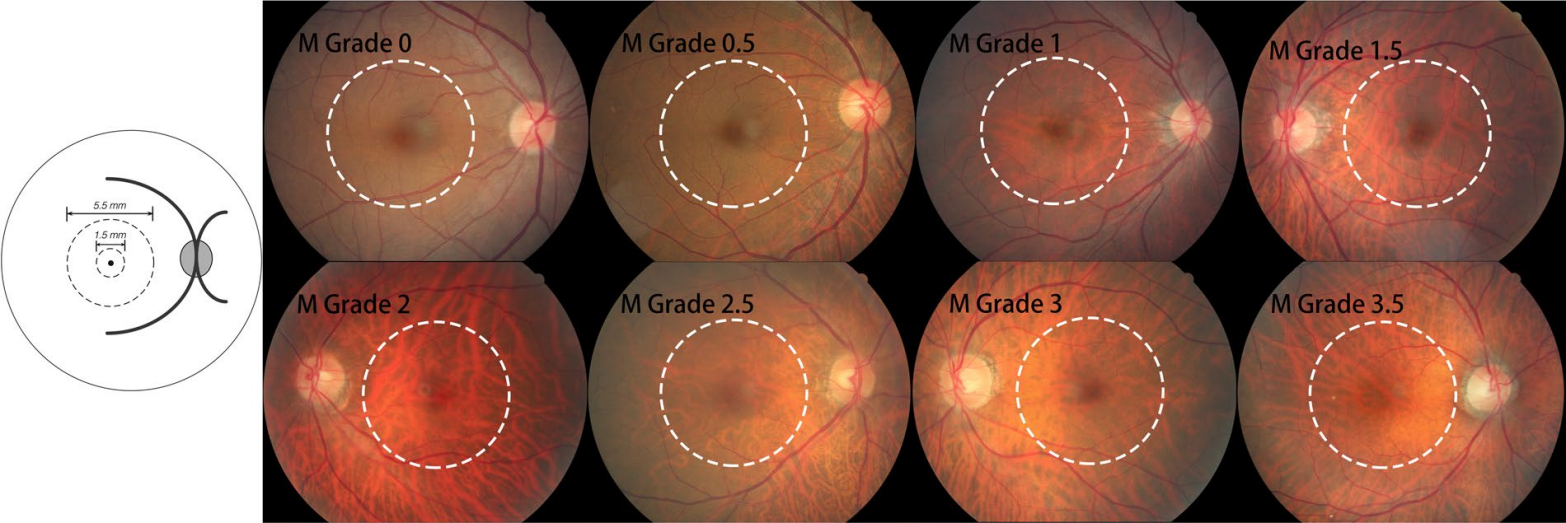
Subregions and feature photographs of macular fundus tessellation as defined by the ophthalmoscopic visibility of the large choroidal vessels in the macular region.

**Figure 2:**
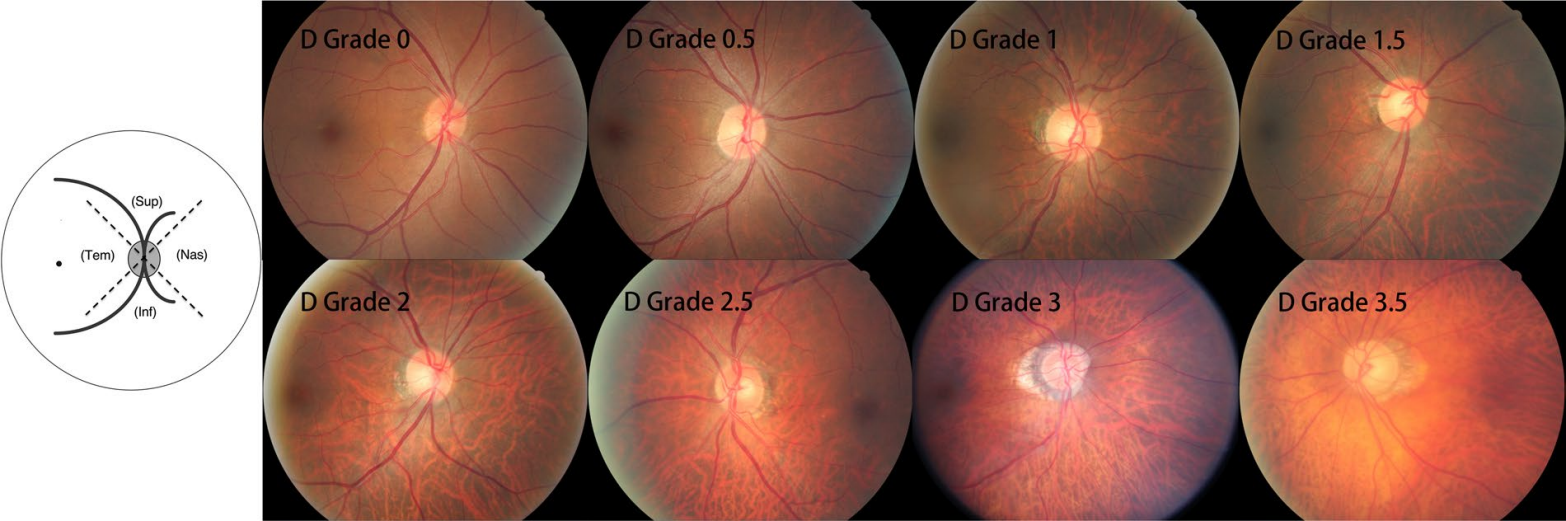
Subregions and feature photographs of fundus tessellation as defined by the ophthalmoscopic visibility of the large choroidal vessels in the peripapillary region. Inf = inferior; Nas = nasal; Sup = superior; Tem = temporal.

Using the color fundus photographs, fundus tessellation was graded as described recently^9^. The degree of fundus tessellation was assessed on the 45° fundus photographs centered on the macula and on the optic nerve head. The macular region was divided into the central macula zone and the perimacular zone, and the peripapillary area was divided into 4 quadrants (superior, nasal, inferior, and temporal). In each zone, fundus tessellation was determined and the mean value for the macular region and for the peripapillary region was taken for further statistical analysis. Fundus tessellation was differentiated between grade 0 and grade 3 based on the visibility of the large choroidal vessels (Figs 1 and 2). In assessing the photographs, the contrast, brightness, background pigmentation and photographic quality of the eyes were taken into account. Standard photographs were used as reference to re-calibrate the subjective assessment of the photographs during the examinations. The assessment of the fundus tessellation was performed as a single examination independently of other examinations. It was carried out by a trained examiner (YNY.), regularly supervised by a panel of experienced ophthalmologists (YXW, JBJ). The reproducibility of the technique was assessed in a previous investigation^9^. The images of 100 eyes of 100 participants had randomly been selected and had been assessed twice by a trained grader (YNY) in a masked manner with an interval of two weeks between both examinations. It revealed kappa values higher than 0.80 for the re-assessment of fundus tessellation in the four peripapillary regions and in the macular area. A change in fundus tessellation was calculated as the difference between the degree of fundus tessellation in 2011 minus the degree of fundus tessellation in 2001.

For the statistical analysis, we used a commercially available software package (SPSS for Windows, version 22.0, IBM-SPSS, Chicago, IL). We calculated the mean values (presented as mean standard deviation) of the main parameters. The associations between the progression of fundus tessellation and the other ocular or systemic parameters were assessed first in a univariate analysis. A subsequent multivariate analysis included the progression of fundus tessellation as the dependent variable and all those parameters as independent variables that were significantly associated with the progression of fundus tessellation in the univariate analysis. We then dropped out of the list of the independent variables those parameters that either showed a high collinearity or which were no longer significantly associated with the fundus tessellation progression. We presented the standardized regression coefficient beta, the non-standardized regression coefficient B and the 95% confidence intervals (CIs). All *P*-values were two-sided and considered statistically significant when the values were <0.05.

## Results

Out of 4439 individuals participating in the baseline examination in 2001, 379 (8.5%) individuals died in the 10-year period. From the remaining 4060 subjects, 2695 (66.4%) individuals were re-examined in 2011. Out of these 2695 individuals, fundus photographs obtained in 2001 and in 2011 were available for 4846 eyes of 2466 (91.5%) subjects (1052 (43.3%) men). The mean age of these participants was 64.1 ± 9.4 years (median: 63.0 years; range: 50–91 years) in 2011, the mean refractive error was −0.19 ± 2.08 diopters (median: +0.25 diopters; range: −22.0 to +7.00 diopters) and the mean axial length was 23.3 ± 1.1 mm (median: 23.1 mm; range: 18.96–30.88 mm). An assessment of the progression of fundus tessellation was not possible, if the fundus photographs taken in 2001 or 2011 could not be evaluated due to opacities of the optic media of the eye or due to a dilatation lag of the pupil. The group of individuals without assessment of fundus tessellation progression compared with the group of individuals with assessment of fundus tessellation progression was significantly older (69.9 ± 10.7 years versus 64.1 ± 9.4 years; *P* < 0.001) and showed a higher proportion of men (43.8%/56.2% versus 43.3%/56.7%; *P* < 0.001). Both groups did not differ significantly in axial length (23.2 ± 1.3 vs. 23.3 ± 1.1; *P* = 0.59).

The mean progression of fundus tessellation progression in the macular region was 0.18 ± 0.37 grades (median, 0.00; range, 0.0 to +2.5) and the mean degree of progression of fundus tessellation in the peripapillary region was 0.19 ± 0.26 (median, 0.13; range, −0.38 to +2.00). A decrease in the peripapillary fundus tessellation was detected in 4 (0.1%) of the eyes examined. Any increase in the degree of the peripapillary fundus tessellation was detected in 2795 (57.7%) eyes, and an increase by more than 0.25 degree was found in 1050 (21.7%). Any increase in the degree of the macular fundus tessellation was detected in 1158 (23.9%) eyes.

The progression in the macular region and the progression in the peripapillary region were significantly associated with each other (correlation coefficient r: 0.61; *P* < 0.001). The progression of the peripapillary fundus tessellation was significantly (*P* < 0.001) the highest in the temporal peripapillary region (0.25 ± 0.45 grades), followed by the inferior peripapillary region (0.21 ± 0.38 grades; *P* < 0.001) and the nasal peripapillary region (0.17 ± 0.31 grades), where it was significantly higher (*P* < 0.001) than in the superior peripapillary region (0.14 ± 0.28 grades).

In univariate analysis, a more marked fundus progression of tessellation in the macular region was associated with the systemic parameters of older age (*P* < 0.001), urban region of habitation (*P* < 0.001), higher level of education (*P* < 0.001), lower cognitive score (*P* < 0.001), lower consumption of alcohol (*P* < 0.001), lower prevalence of ever smoking (*P* < 0.001), lower number of cigarette package years (*P* = 0.03), and with other systemic and ocular parameters (Table 1). The multivariate analysis included the degree of macular fundus tessellation progression as the dependent variable. Independent variables were all those parameters that were significantly associated with the progression of fundus tessellation in the univariate analysis. Out of the list of independent parameters, we dropped In a step-by-step manner parameters either due to relatively high collinearity or due to missing statistical significance.

**Table 1:** Associations (univariate analysis) between the 10-year progression of macular fundus tessellation and systemic and ocular parameters in the Beijing Eye Study 2001–2011.

Parameter	*P*-Value	Standardized Regression Coefficient beta	Non-standardized Regression Coefficient B	95% Confidence Interval
Systemic Parameters				
Age (Years)	<0.001	0.37	0.02	0.01, 0.02
Rural/Urban Region of Habitation	<0.001	0.22	0.17	0.15, 0.19
Body Height (cm)	0.002	−0.05	−0.002	−0.003, −0.001
Body Weight (kg)	<0.001	−0.10	−0.003	−0.004, −0.002
Body Mass Index (kg/m^2^)	<0.001	−0.08	−0.01	−0.01, −0.01
Level of Education (1–5)	<0.001	0.07	0.03	0.02, 0.04
Self-Reported Income	<0.001	0.16	0.03	0.02, 0.03
Cognitive Score	<0.001	−0.08	−0.009	−0.012, −0.006
Alcohol Consumption Frequency	<0.001	−0.10	−0.02	−0.03, −0.01
Smoking Never/Former/Current	<0.001	−0.08	−0.04	−0.05, −0.02
Smoking Never/Ever	<0.001	−0.06	−0.05	−0.07, −0.02
Smoking Package Years	0.03	−0.04	−0.001	−0.002, 0.000
Physical Activity				
“How Many Days Do you Walk?”	0.03	0.03	0.005	0.000, 0.010
“How Many Hours Do You Do Vigorously Intensive Sport or Activities Per Day?”	0.009	−0.04	−0.015	−0.026, −0.004
“How Many Days Do You Do Moderately Intensive Sport or Activities Per Day?”	<0.001	−0.14	−0.02	−0.02, −0.02
“How Many Hours Do You Sit Per Day?”	0.92			
Quality of Life				
Summed Score	0.003	0.06	0.02	0.01, 0.04
Mobility: I have no/some problems in walking about/I am confined to bed	<0.001	0.09	0.12	0.07, 0.18
Self-Care: I have I have no/some problems in washing or dressing myself/I am unable to wash or dress myself	<0.001	0.09	0.16	0.09, 0.24
Usual Activities (e.g. Work, study, housework, family or leisure activities): I am able to wash or dress myself/I have some problems with performing my usual activities/I am unable to perform my usual activities	<0.001	0.07	0.11	0.05, 0.17
Pain/Discomfort:	0.21			
Anxiety/Depression: I am not/moderately/extremely anxious or depressed	0.04			
Depression Score	0.04	−0.04	−0.05	−0.10, −0.003
Blood Concentration of:				
Glucose (mmol/L)	0.72			
Glycosylated hemoglobin HbA1c	0.33			
High-Density Lipoproteins (mmol/L)	0.62			
Low-Density Lipoproteins (mmol/L)	0.13			
Triglycerides (mmol/L)	0.82			
Cholesterol (mmol/L)	0.36			
C-reactive Protein	0.89			
Creatinine (mmol/L)	<0.001	0.14	0.004	0.002, 0.005
Estimated Glomerular Filtration Rate (GFR) (mL/min/1·73 m²)	<0.001	−0.20	−0.003	−0.004, −0.002
Change in Glucose (mmol/L)	0.75			
Change in High-Density Lipoproteins (mmol/L)	0.41			
Change in Triglycerides (mmol/L)	0.71			
Diabetes Mellitus, Prevalence	0.02	0.06	0.06	0.01, 0.10
Systolic Blood Pressure (mmHg)	0.06	0.03	0.001	0.00, 0.001
Diastolic Blood Pressure (mmHg)	<0.001	−0.11	−0.003	−0.004, −0.002
Mean Blood Pressure (mmHg)	0.001	−0.05	−0.001	−0.002, −0.001
Arterial Hypertension	<0.001	0.07	0.06	0.03, 0.08
Estimated Cerebrospinal Fluid Pressure (mm Hg)	<0.001	−0.27	−0.03	−0.03, −0.03
Ocular Parameters				
Refractive Error (Diopters)	<0.001	−0.10	−0.02	−0.02, −0.01
Axial Length (mm)	<0.001	0.15	0.05	0.04, 0.06
Anterior Corneal Curvature Radius (mm)	0.001	0.07	0.11	0.04, 0.17
Central Corneal Thickness (µm)	<0.001	0.05	0.001	0.000, 0.001
Anterior Chamber Depth (mm)	0.84			
Lens Thickness (mm)	<0.001	0.10	0.12	0.07, 0.16
Intraocular Pressure mmHg)	0.04	−0.03	−0.004	−0.008, 0.000
Retinal Nerve Fiber Layer Thickness (µm)	<0.001	−0.11	−0.003	−0.004, −0.003
Localized Defects of the Retinal Nerve Fiber Layer, Prevalence	<0.001	0.09	0.12	0.08, 0.16
Subfoveal Choroidal Thickness (µm)	<0.001	−0.47	−0.002	−0.002, −0.002
Peripapillary Choroidal Thickness (µm)	<0.001	−0.41	−0.003	−0.003, −0.003
Macular Retinal Thickness (µm)	<0.001	0.10	0.001	0.001, 0.002
Optic Disc Size (mm^2^)	<0.001	0.12	0.09	0.06, 0.12
Optic Cup Size (mm^2^)	0.84			
Neuroretinal Rim Area (mm^2^)	0.63			
Parapapillary Alpha Zone (mm^2^)	0.11			
Parapapillary Beta/Gamma Zone (mm^2^)				
Dry Eye, Yes or No	0.003	0.06	0.05	0.02, 0.08
Dry Eye, Number of Days Per Week	<0.001	0.08	0.01	0.01, 0.02
Keratoconus (Anterior Corneal Curvature refractive Power ≥48 Diopters)	0.07	0.04	0.20	−0.01, 0.41
Pseudoexfoliation Syndrome	0.005	0.06	0.10	0.03, 0.18
Nuclear Cataract	<0.001	0.14	0.11	0.08, 0.13
Cortical Cataract	<0.001	0.14	0.15	0.11, 0.18
Subcapsular Posterior Cataract	<0.001	0.12	0.24	0.17, 0.30
Glaucoma, Prevalence, Total	<0.001	0.09	0.15	0.10, 0.19
Open-Angle Glaucoma	0.005	0.04	0.09	0.03, 0.15
Primary Angle-Closure Glaucoma	<0.001	0.06	0.21	0.11, 0.32
Age-Related Macular Degeneration, Prevalence, Total	0.17			
Age-Related Macular Degeneration, Early Stage	0.25			
Age-Related Macular Degeneration, Intermediate Stage	0.39			
Age-Related Macular Degeneration, Late Stage	0.61			
Diabetic Retinopathy, Prevalence	0.60			
Diabetic Retinopathy, Level	0.26			
Retinal Vein Occlusion, Total	0.94			
Branch Retinal Vein Occlusion	0.03	0.04	0.11	0.01, 0.21
Central Serous Choroidopathy	0.95			

In the final model, a higher progression of macular fundus tessellation was associated (correlation coefficient r: 0.53) with older age (*P* < 0.001), worse best corrected visual acuity (*P* = 0.001), thinner subfoveal (*P* < 0.001) and peripapillary choroidal thickness in 2011 (*P* = 0.003), thicker retinal nerve fiber layer thickness (*P* = 0.004), higher intraocular pressure (*P* = 0.03), higher level of education (*P* = 0.003) and more myopic change in refractive error (*P* < 0.001) (Table 2). If we additionally performed a Bonferroni correction to correct for performing multiple comparisons in the multivariate analysis, the associations between increased macular fundus tessellation and older age, worse best corrected visual acuity, thinner subfoveal and peripapillary choroidal thickness in 2011, thicker retinal nerve fiber layer thickness, higher level of education and more myopic change in refractive error (*P* < 0.001) remained statistically significant, while intraocular pressure was no longer significantly correlated with the change in fundus tessellation.

**Table 2:** Associations (multivariate analysis) between 10-year progression of macular fundus tessellation and systemic and ocular parameters in the Beijing Eye Study 2001–2011.

Parameter	*P*-Value	Standardized Regression Coefficient beta	Non-standardized Regression Coefficient B	95% Confidence Interval	Variance Inflation Factor
Age (Years)	<0001^*^	0.18	0.007	0.006, 0.009	1.60
Best Corrected Visual Acuity (logMAR)	0.001^*^	0.08	0.28	0.12, 0.44	1.51
Subfoveal Choroidal Thickness (µm)	<0.001*	−0.32	−0.001	−0.002, −0.001	2.35
Peripapillary Choroidal Thickness (µm)	0.003^*^	−0.08	−0.001	−0.001, 0.000	2.22
Retinal Nerve Fiber Layer Thickness (µm)	0.004^*^	0.06	0.002	0.001, 0.003	1.09
Intraocular Pressure (mmHg)	0.03	0.04	0.006	0.000, 0.011	1.05
Level of Education	0.003^*^	0.06	0.02	0.007, 0.036	1.09
Change in Refractive Error (2011 minus 2001)	<0.001^*^	−0.08	−0.03	−0.04, −0.01	1.09

^*^Statistically significant associations after performing a Bonferroni correcting for conducting multiple comparisons.

If the peripapillary choroidal thickness was dropped due to a relatively high collinearity (variance inflation factor: 2.22) and the cognitive function score was added to the model, the association between the subfoveal choroidal thickness and the progression of fundus tessellation became stronger (beta: −0.37), while intraocular pressure, retinal nerve fiber layer thickness (*P* = 0.06) and best corrected visual acuity (*P* = 0.08) were no longer significantly associated. In the resulting model, a more marked progression of fundus tessellation was correlated with older age (*P* < 0.001), thinner subfoveal choroidal thickness (*P* < 0.001), higher level of education (*P* < 0.001), more myopic change in refractive error (*P* < 0.001) and lower cognitive function (*P* = 0.02) (Table 3). If the subfoveal choroidal thickness was dropped from the model, an increase in macular fundus tessellation was strongly (*P* < 0.001) associated with a decrease in the cognitive function score (beta: −0.07; B: −0.007; 95%CI: −0.011, −0.004). If axial length was added to the model, it was not significantly associated (*P* = 0.73).

**Table 3:** Associations (multivariate analysis) between 10-year progression of macular fundus tessellation and systemic and ocular parameters in the Beijing Eye Study 2001–2011, after dropping peripapillary choroidal thickness.

Parameter	*P*-Value	Standar-dized Regression Coefficient beta	Non-standardized Regression Coefficient B	95% Confidence Interval	Variance Inflation Factor
Age (Years)	<0001	0.22	0.009	0.007, 0.010	1.27
Subfoveal Choroidal Thickness (µm)	<0.001	−0.37	−0.001	−0.001, −0.001	1.22
Level of Education	<0.001	0.09	0.03	0.02, 0.05	1.45
Cognitive Function Score	0.02	−0.05	−0.006	−0.011, −0.001	1.52
Change in Refractive Error (2011 minus 2001)	<0.001	−0.09	−0.03	−0.04, −0.02	1.04

If progression of macular fundus tessellation was defined as any increase in fundus tessellation, a binary regression analysis revealed that a higher prevalence of a macular fundus tessellation progression was associated with older age (*P* < 0.001; OR: 1.04; 95%CI: 1.03, 1.06), thinner subfoveal (*P* < 0.001; OR: 0.99; 95%CI: 0.99, 0.99) and peripapillary choroidal thickness in 2011 (*P* < 0.001; OR: 0.99; 95%CI: 0.99, 0.99), thicker retinal nerve fiber layer thickness (*P* = 0.004; OR: 1.02; 95%CI: 1.01, 1.03), higher intraocular pressure (*P* = 0.02; OR: 1.05; 95%CI: 1.01, 1.10), higher level of education (*P* = 0.02; OR: 1.14; 95%CI: 1.02, 1.28) and more myopic change in refractive error (*P* = 0.001; OR: 0.88; 95%CI: 0.79, 0.98).

Increase in peripapillary fundus tessellation was significantly correlated with older age (*P* < 0.001), worse best corrected visual acuity (*P* = 0.001), thinner peripapillary choroidal thickness in 2011 (*P* < 0.001), higher prevalence of ever smoking (*P* = 0.001), and more myopic change in refractive error (*P* < 0.001) (Table 4).

**Table 4:** Associations (multivariate analysis) between 10-year progression of peripapillary fundus tessellation and systemic and ocular parameters in the Beijing Eye Study 2001–2011.

Parameter	*P*-Value	Standardized Regression Coefficient beta	Non-standardized Regression Coefficient B	95% Confidence Interval	Variance Inflation Factor
Age (Years)	<0001	0.20	0.006	0.004, 0.007	1.47
Best Corrected Visual Acuity (logMAR)	0.001	0.07	0.19	0.08, 0.30	1.40
Peripapillary Choroidal Thickness (µm)	<0.001	−0.35	−0.002	−0.002, −0.002	1.17
Smoking Ever	0.004	0.05	0.03	0.01, 0.05	1.01
Change in Refractive Error (2011 minus 2001)	<0.001	−0.07	−0.02	−0.03, −0.009	1.07

If progression of peripapillary fundus tessellation was defined as an increase in peripapillary fundus tessellation by more than 0.25 degree, a prevalence of a progression of peripapillary fundus tessellation was correlated with older age (*P* < 0.001; OR: 1.05; 95%CI: 1.04, 1.06), thinner peripapillary choroidal thickness (*P* < 0.001; OR: 0.98; 95%CI: 0.98, 0.98), and higher prevalence of ever smoking (*P* = 0.003; OR: 1.46; 95%CI: 1.14, 1.86).

## Discussion

This longitudinal population-based study was conducted to assess whether changes in the degree of fundus tessellation, considered as a surrogate for subfoveal choroidal thickness and as observed in a 10-year period, were associated with the prevalence of ocular disorders and systemic parameters such as cognitive function and smoking. It revealed that an increase in macular fundus tessellation was associated with a lower cognitive function, after adjusting for thinner choroidal thickness in 2011, older age, myopic change in refractive error, and higher level of education. An increase in peripapillary fundus tessellation was additionally correlated with a higher prevalence of ever smoking. The findings point to the potential clinical value of the assessment of fundus tessellation as an ophthalmoscopical surrogate of the posterior choroidal thickness. Potential associations between lower cognitive function and an increase in fundus tessellation/decrease in choroidal thickness as well as between smoking and progression of fundus tessellation/progressive choroidal thinning may be further evaluated.

The findings obtained in our study are partially consistent with the results of a previous cross-sectional investigation in which a higher degree of fundus tessellation was associated with older age, male sex, lower body mass index, worse best corrected visual acuity, thinner subfoveal choroidal thickness, longer axial length, larger parapapillary beta/gamma zone, lower prevalence of intermediate age-related macular degeneration, and lower prevalence of late age-related macular degeneration^9^. In agreement with the previous investigation, the present study found a significant correlation between a larger increase in macular fundus tessellation and older age, thinner subfoveal choroidal thickness, and more marked myopic increase in refractive error. As compared to the previous cross-sectional analysis, the present longitudinal investigation did not show associations between a change in macular fundus tessellation and gender, body mass index, axial length, size of parapapillary beta/gamma zone and prevalence of intermediate and late age-related macular degeneration.

The association between a larger increase in fundus tessellation and lower cognitive function, as assessed in multivariate analysis, agrees with the result of a previous study in which thinner subfoveal choroidal thickness was correlated with a lower cognitive function score in a multivariate analysis^22^. In a reciprocal manner, lower cognitive function score was correlated with thinner subfoveal choroidal thickness in multivariate analysis. In the previous investigation as well as in the present study, the association between choroidal thickness, or fundus tessellation, and cognitive function remained significant after adjusting for best corrected visual acuity. It suggested that a potential association between lower visual acuity and lower cognitive function could not explain the relationship between thinner choroidal thickness and lower cognitive function.

The relationship between a larger increase in macular fundus tessellation and a larger increase in refractive error corresponds with the strong association between the degree of fundus tessellation and axial length, which is a defining and causative characteristic of refractive error^9,23,24^. The association between the fundus tessellation and a thin posterior choroid is in agreement with earlier studies by Spaide and colleagues in which eyes with an abnormally thin choroid showed a marked tessellated fundus^2^. In a study by Yoshihara and coworkers a higher degree of fundus tessellation was correlated with thinner subfoveal and nasal choroidal thickness in 100 healthy Japanese^7^. The increase in fundus tessellation with older age reflects the association between thinner posterior choroidal thickness and older age in cross-sectional analysis^23^.

Interestingly, an increase in the peripapillary fundus tessellation was additionally associated with a higher frequency of smoking. Sigler and colleagues demonstrated an association between a history of cigarette smoking and a thinner choroid in patients with an age of 65+ years and with early age-related macular degeneration as compared to individuals of a control group^25^. The finding of an association between smoking and a thinner choroid is also supported by the study performed by Moschos and associates^26^. These findings may suggest a negative effect of smoking on the choroidal vasculature.

When discussing the results of our study, its limitations should be noted. First, the assessment of fundus tessellation as a subjective and semi-quantitative method was dependent on the skills of the examiner. However, the examiner in our study was trained and regularly supervised by a panel of experienced ophthalmologists. Also, standard photographs were used to re-calibrate the assessment of the photographs during the series of examinations. Second, the participation rate in our survey was 66.4% of the survivors after a 10-year period. This figure was lower than the participation rate in the 10-year follow-up survey of the Blue Mountains Eye Study (75.6% of survivors) and in the Beaver Dam Eye Study (82.9% of survivors). The non-participation in our study might have influenced the results of the investigation. The reason for the lower follow-up response in the current study was the relatively high mobility of the population in Greater Beijing, with a substantial number of inhabitants having moved away during the follow-up period. Since the reason to move (e.g. construction of a new airport) might have been independent of the general health condition of the individual, it might not have induced a major bias into the study. Third, the appearance of the fundus tessellation depends on the physiological background pigmentation of the eye, and thus on the ethnic background. The results of our study obtained in Chinese may therefore not directly be transferred on other ethnicities. Fourth, the subfoveal choroidal thickness was taken as a surrogate for the macular choroidal thickness, while the macular fundus tessellation was graded over the whole area of the macula. Since choroidal thickness measurements can vary between various locations in the macular region, it would have been more precise assessing the choroidal thickness at various locations in the macular region. Fifth, the present study is limited to a correlation analysis. Since various parameters such as choroidal thickness were not measured at the baseline examination in 2001, the study could not prove causal relationships between the changes in fundus tessellation and the other parameters assessed. Sixth, although fundus tessellation was strongly associated with choroidal thickness in the previous cross-sectional investigation, the mean magnitude of progression of fundus tessellation in the present follow-.up study was in the relatively small order of 0.2 on the 0–3 point categorical scale. It may suggest that clinically, fundus tessellation would have greater utility as a yes/no variable rather than a method for estimating choroidal thickness. Seventh, we tested in the univariate analysis potential associations between fundus tessellation and more than 50 variables (Table 1). These variables included most of the parameters primarily tested in the Beijing Eye Study. They were not selected based on an a priori knowledge that they were correlated with fundus tessellation but they were selected since we wanted to examine the strength of associations already known to be correlated with fundus tessellation in a multivariate and longitudinal manner, and we wanted to find new, yet unknown, associations of fundus tessellation. To avoid the risk of a statistical type 1 error, we then performed the multivariate analysis which takes into account correlations between the independent parameters. Correspondingly, we checked for collinearity between the independent parameters in the multivariate analysis. Furthermore, we eventually performed a Bonferroni correction of the results of the multivariate analysis to additionally correct for conducting multiple comparisons.

We conducted this study to assess the change in fundus tessellation during a study period of 10 years and to examine its associations with other ocular and systemic parameters. The clinical value of the parameter of fundus tessellation is its strong association with a decreased choroidal thickness, so that a marked fundus tessellation can be regarded as a surrogate for a leptochoroid. Besides the associations mentioned above and listed in Table 2, we did not detect correlations between a change in fundus tessellation and diseases such as angle-closure glaucoma or age-related macular degeneration (Table 2). One of the reasons for the lack of such correlations may be that some of the diseases which are associated with an abnormal choroidal thickness such as central serous choroidopathy, Vogt-Koyanagi-Harada’s disease, polypoidal choroidal vasculopathy and idiopathic choroidal neovascularization, have a relatively low prevalence so that they were either not present in the study population or their prevalence was too low for a meaningful multivariate statistical analysis. An increase in fundus tessellation was however correlated with an increase in myopic refractive error fitting with the correlation between increasing axial myopia and choroidal thinning.

In conclusion, an increase in macular fundus tessellation, observed during a ten-year follow-up, was a surrogate for thinning of the posterior choroid and it was associated with lower cognitive function, after adjusting for choroidal thickness, age, change in refractive error and educational level. An increase in the peripapillary fundus tessellation was additionally correlated with a higher prevalence of ever smoking. The findings indicate the clinical value of fundus tessellation as an ophthalmoscopical surrogate of the posterior choroidal thickness and they suggest potential associations between a lower cognitive function and an increase in fundus tessellation/decrease in choroidal thickness as well as between smoking and progression of fundus tessellation or progressive choroidal thinning.

## References

[1] Jonas JB, Gründler A (1996). Optic disc morphology in ‘age-related atrophic glaucoma’. Graefes Arch. Clin. Exp. Ophthalmol..

[2] Spaide RF (2009). Age-related choroidal atrophy. Am. J. Ophthalmol..

[3] Chen H (2012). The types and severity of high myopic maculopathy in Chinese patients. Ophthalmic Physiol. Opt..

[4] Switzer DW, Mendonça LS, Saito M, Zweifel SA, Spaide RF (2012). Segregation of ophthalmoscopic characteristics according to choroidal thickness in patients with early age-related macular degeneration. Retina..

[5] Warrow DJ, Hoang QV, Freund KB (2013). Pachychoroid pigment epitheliopathy. Retina..

[6] Koh VT (2013). Pathologic changes in highly myopic eyes of young males in Singapore. Ann. Acad. Med. Singapore..

[7] Yoshihara N, Yamashita T, Ohno-Matsui K, Sakamoto T (2014). Objective analyses of tessellated fundi and significant correlation between degree of tessellation and choroidal thickness in healthy eyes. PLoS One..

[8] Ohno-Matsui K (2015). International classification and grading system for myopic maculopathy. Am. J. Ophthalmol..

[9] Yan YN (2015). Fundus tessellation: Prevalence and associated factors. The Beijing Eye Study 2011. Ophthalmology..

[10] Imamura Y, Fujiwara T, Margolis R, Spaide RF (2009). Enhanced depth imaging optical coherence tomography of the choroid in central serous chorioretinopathy. Retina..

[11] Chung SE, Kang SW, Lee JH, Kim YT (2011). Choroidal thickness in polypoidal choroidal vasculopathy and exudative age-related macular degeneration. Ophthalmology..

[12] Xu J (2013). Subfoveal choroidal thickness in diabetes and diabetic retinopathy. The Beijing Eye Study 2011. Ophthalmology..

[13] Wang YX (2014). Subfoveal choroidal thickness in glaucoma. The Beijing Eye Study 2011. PLoS One..

[14] Spaide RF, Koizumi H, Pozzoni MC (2008). Enhanced depth imaging spectral-domain optical coherence tomography. Am. J. Ophthalmol..

[15] Margolis R, Spaide RF (2009). A pilot study of enhanced depth imaging optical coherence tomography of the choroid in normal eyes. Am. J. Ophthalmol..

[16] Ikuno Y, Tano Y (2009). Retinal and choroidal biometry in highly myopic eyes with spectral-domain optical coherence tomography. Invest. Ophthalmol. Vis. Sci..

[17] Ojima Y (2009). Improved visualization of polypoidal choroidal vasculopathy lesions using spectral-domain optical coherence tomography. Retina..

[18] Dhoot DS (2013). Evaluation of choroidal thickness in retinitis pigmentosa using enhanced depth imaging optical coherence tomography. Br. J. Ophthalmol..

[19] Lin P (2012). Image inversion spectral-domain optical coherence tomography optimizes choroidal thickness and detail through improved contrast. Invest. Ophthalmol. Vis. Sci..

[20] Folstein MF, Folstein SE, McHugh PR (1975). “Mini-mental state”. A practical method for grading the cognitive state of patients for the clinician. J. Psychiatr. Res..

[21] Liu HH (2010). Prevalence and progression of myopic retinopathy in Chinese adults: The Beijing Eye Study. Ophthalmology..

[22] Jonas JB (2016). Cognitive function and subfoveal choroidal thickness. The Beijing Eye Study. Ophthalmology..

[23] Wei WB (2013). Subfoveal choroidal thickness: the Beijing Eye Study. Ophthalmology..

[24] Fujiwara T, Imamura Y, Margolis R, Slakter JS, Spaide RF (2009). Enhanced depth imaging optical coherence tomography of the choroid in highly myopic eyes. Am. J. Ophthalmol..

[25] Sigler EJ, Randolph JC, Calzada JI, Charles S (2014). Smoking and choroidal thickness in patients over 65 with early-atrophic age-related macular degeneration and normals. Eye (Lond)..

[26] Moschos MM, Nitoda E, Laios K, Ladas DS, Chatziralli IP (2016). The impact of chronic tobacco smoking on retinal and choroidal thickness in Greek population. Oxid. Med. Cell Longev..

